# Multicolor 4D printing of shape-memory polymers for light-induced selective heating and remote actuation

**DOI:** 10.1038/s41598-020-63020-9

**Published:** 2020-04-10

**Authors:** Hoon Yeub Jeong, Byung Hoon Woo, Namhun Kim, Young Chul Jun

**Affiliations:** 1 School of Materials Science and Engineering, Ulsan National Institute of Science and Technology (UNIST), Ulsan, 44919 Republic of Korea; 2 School of Mechanical, Aerospace and Nuclear Engineering, UNIST, Ulsan, 44919 Republic of Korea

**Keywords:** Engineering, Mechanical engineering

## Abstract

Four-dimensional (4D) printing can add active and responsive functions to three-dimensional (3D) printed objects in response to various external stimuli. Light, among others, has a unique advantage of remotely controlling structural changes to obtain predesigned shapes. In this study, we demonstrate multicolor 4D printing of shape-memory polymers (SMPs). Using color-dependent selective light absorption and heating in multicolor SMP composites, we realize remote actuation with light illumination. We experimentally investigate the temperature changes in colored SMPs and observe a clear difference between the different colors. We also present simulations and analytical calculations to theoretically model the structural variations in multicolor composites. Finally, we consider a multicolor hinged structure and demonstrate the multistep actuation by changing the color of light and duration of illumination. 4D printing can allow complex, multicolor geometries with predesigned responses. Moreover, SMPs can be reused multiple times for thermal actuation by simply conducting thermomechanical programming again. Therefore, 4D printing of multicolor SMP composites have unique merits for light-induced structural changes. Our study indicates that multicolor 4D printing of SMPs are promising for various structural changes and remote actuation.

## Introduction

Three-dimensional (3D) printing is appropriate for the fabrication of complex multi-material structures that are difficult to realize using conventional methods. 3D printing is being widely used in many different technical areas^1–7^. However, in most cases, 3D-printed structures remain static as printed. A new concept, known as four-dimensional (4D) printing, has been proposed by Tibbits *et al*.^8^. They developed active composite structures consisting of swelling and rigid materials. Using water-swelling materials, flat 3D-printed structures can be transformed into predesigned 3D shapes. 4D printing can add active, responsive functions to 3D-printed objects in various ways^9^. For example, 4D printing research has been conducted involving hydrogels^10–12^ or shape-memory polymers (SMPs)^13–15^. These materials can respond to external stimuli such as heat, humidity, pH value, and light^16–18^. Using the swelling and contraction of hydrogels in water, it is possible to realize biomimetic 3D-printed structures^19^. Although a reversible shape change is allowed for hydrogels, it is often very slow (taking several minutes)^20^ and working only in limited environments (e.g., in a solvent). In contrast, SMPs allow for a rapid shape change of 3D-printed objects with large structural deformation. SMPs can be deformed into arbitrary temporary shapes and the temporary shape can be fixed during a glass-transition or crystallization process. After this thermomechanical programming, SMPs can recover their original shapes when heated above a transition temperature (the glass-transition temperature, T_g_, in our case). The T_g_ for SMPs can be adjusted via chemical synthesis or by simply mixing two SMPs in a different ratio^21^ during 3D printing (called digital SMPs). Moreover, SMPs are rigid enough to bear loadings and can be reused for structural changes and actuation; the programming and recovery processes can be repeated multiple times. Owing to these unique features, many researchers have utilized SMPs in 4D printing studies. Several of these studies have focused on sequential shape changes. Mao *et al*. fabricated a sequential self-folding structure using digital SMPs with different values of T_g_^22^. Wu *et al*. proposed the multi-shape memory behavior of 3D-printed SMP composites and these structures were activated in water at different temperatures^23^.

Light has an advantage in that it can induce stimuli-responsive structural changes remotely. Although magnetic fields can also allow remote actuation^24–26^, the allowed length could be limited to locate objects close to a magnet. The magnetic fields for actuation can be also generated from electric coils that can be integrated into actuators. However, this can complicate actuator design and increase the overall weight. Several studies on light-induced shape changes have recently been conducted, although they do not involve 3D printing. Mu *et al*. fabricated light-activated polymer-laminated composites^27^. A photochemical reaction with ultraviolet (UV) light induced the bending of composite structures, although the structural changes took a few minutes to occur. Pre-strained polymer sheets (such as Shrinky-Dink) and inkjet printing were also used to create self-folding structures that were activated under light illumination^28,29^. Sequential folding was also demonstrated with different colors of light^30^. Although these are very exciting demonstrations, pre-strained sheets cannot be reused after deformation. Moreover, background heating is often desirable because of the high response temperature (approximately 100 °C).

Inspired by 4D printing of active composites^15,23,31^, we present multicolor 4D printing of SMPs in this paper. Using color-dependent light absorption, we realize remotely controlled shape changes. We study the bending behavior of our multicolor sample via both experiments and theoretical modeling. In contrast to previous studies that involved heating an entire structure, we induce selective heating in our multicolor SMP composites by properly choosing the color of light. This leads to color-dependent structural changes into different shapes. 4D printing can allow the complex geometries of multicolor composites with predesigned responses. In addition, SMPs can be reused multiple times by conducting thermomechanical programming again. Therefore, multicolor 4D printing of SMPs can offer unique merits for light-induced structural changes and remote actuation.

## Results and Discussion

**Figure 1:**
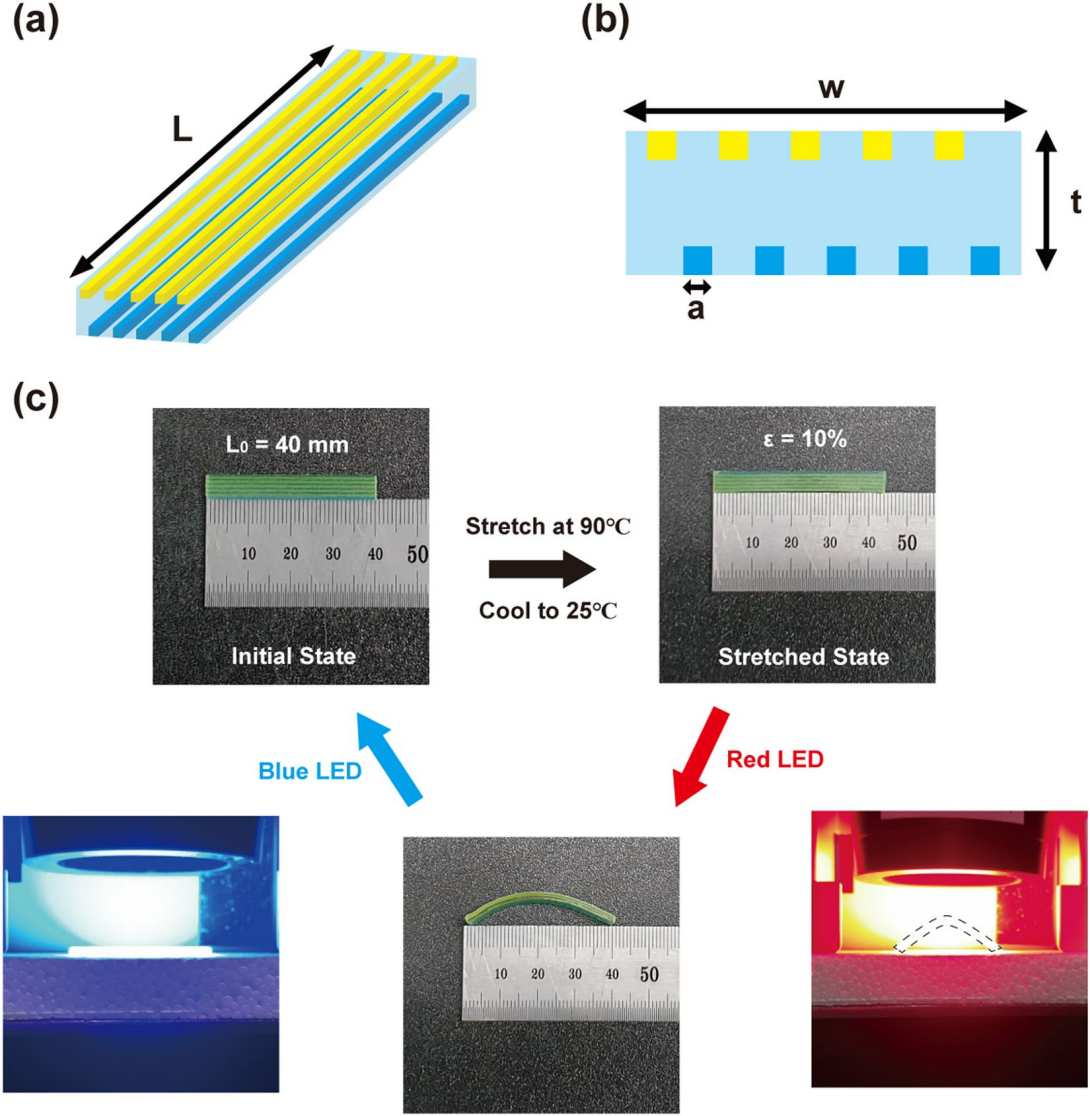
A 3D printed light-activating structure. (**a**) Schematic for the multicolor SMP structure. (**b**) Side view of the structure. (**c**) Thermomechanical programming and bending behavior (the dotted line in the figure is an eye guide).

Figure 1a,b present our light-activating structure (L = 40 mm, w = 5.5 mm, t = 2 mm, a = 0.4 mm). It consists of three materials that are available in the commercial multi-material 3D printer (Stratasys, J750). The yellow (Veroyellow) and blue (Verocyan) fibers are digital SMPs, whereas the sky-blue matrix is a rubber-like transparent material (Tango + ). The yellow and blue fibers are positioned such that incident light can reach both SMPs. The polyJet process (material jetting) was used for the 3D printing of this multicolor composite structure, where photopolymer-ink droplets were jetted and cured with UV light. This polyJet process can create fine features with a resolution of approximately 50 μm in the plane and 15 μm in thickness. The geometric design was first created using computer-aided design (CAD) software and then imported into our 3D printer. Figure 1c demonstrates the thermomechanical programming and light activation in our multicolor structure. First, the entire structure was immersed in hot water and stretched at 90 °C. Then, it was cooled and made rigid by immersing it in cold water (at 25 °C). The stretched structure with a strain of ε = 10% was first illuminated by a red light-emitting diode (LED), and the entire structure was bent downward as shown in Fig. 1c. Then, the bent structure was illuminated by a blue LED, and it reverted to its original flat shape.

**Figure 2:**
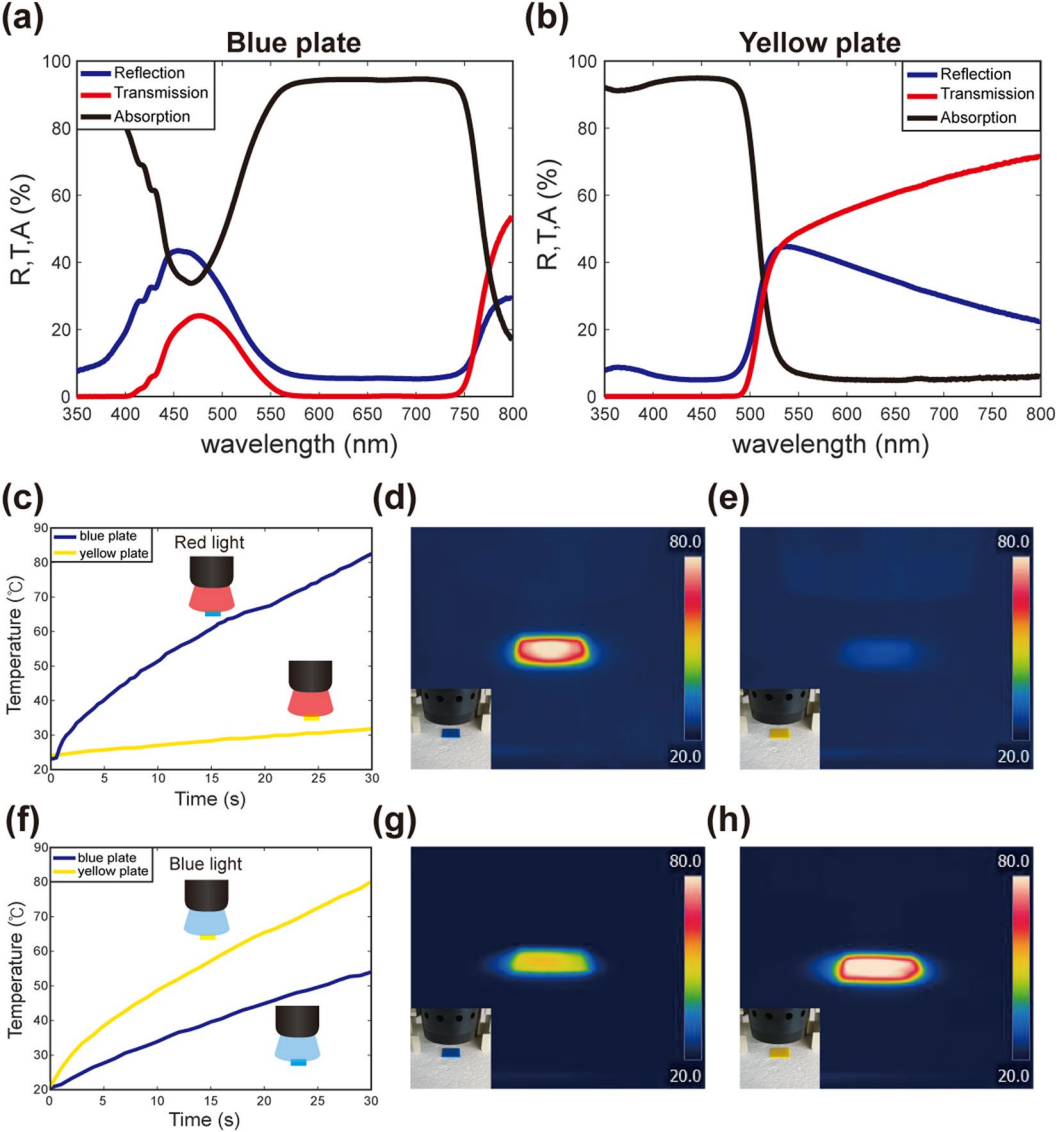
Selective heating of colored SMPs. (**a**,**b**) The reflection, transmission, and absorption spectra of the blue and yellow SMP plates, respectively, in the visible wavelength region. (**c**) The measured temperature change of blue and yellow SMP samples under red LED illumination. (**d**,**e**) IR camera images of the blue and yellow SMP plates, respectively, after 30 seconds of red LED illumination. (**f**) The measured temperature change of blue and yellow SMP plates under blue LED illumination. (**g**,**h**) IR camera images of the blue and yellow SMP plates, respectively, after 30 seconds of blue LED illumination. The dominant wavelength of the red LED was 623 nm, while that of the blue LED was 445 nm. The red LED had a power density of 1.1 mW/mm^2^, while the blue LED had 2.1 mW/mm^2^ measured at a distance of 200 mm.

To understand the observed behavior, we first measured the absorption spectra of printed color plates; refer to Fig. 2a,b. 3D printing of blue and yellow plates with dimensions 25 mm × 25  mm × 1 mm was performed using the same digital SMPs shown in Fig. 1. The transmission (T) and reflection (R) spectra for the two colored plates under normal incidence of light were measured using a spectrophotometer (Cary 5000, Agilent). Then, the absorption (A) spectra were calculated as follows: A = 1 − T − R. Figure 2a,b present the measured spectra for the blue and yellow plates, respectively. We can see that the blue plate absorbs red light strongly (A ~ 95%), while the yellow plate hardly absorbs red light (A ~ 5%) (refer to the black lines in Fig. 2a,b). In the shorter wavelength region, the yellow plate strongly absorbs blue light (A ~ 95%), while the blue plate absorbs blue light moderately (A ~ 41% at 445 nm).

We also directly measured the temperature changes of the color plates under LED illumination using an infrared (IR) imaging camera (FLIR-C2, FLIR) (refer to Fig. 2c–h). A 3-mm-thick piece of styrofoam was placed below the colored plates for thermal insulation from the floor during light illumination. As expected from the absorption spectra, the thermal IR images indicated a clear difference between the two plates under red-LED illumination; refer to Fig. 2d–e. The IR images were recorded over time, and the temperature changes as a function of time are plotted and given in Fig. 2c. The temperature of the blue plate increased rapidly; however, the temperature of the yellow plate exhibited only a slight increase. The temperature of the blue plate increased to 80 °C in 30 s (i.e., above the glass-transition temperature T_g_ of the blue SMP, which is about 66.7 °C). In contrast, the temperature of the yellow plate remained around 30 °C after 30 s. This considerable difference in their temperatures implies that we can selectively heat the blue SMP fibers with red-light illumination. We obtained the same IR images for blue-light illumination; refer to Fig. 2f–h. In this case, the temperature difference between the two plates was smaller because both the plates can absorb blue light to some extent. However, there was still an evident difference in their temperatures.

The difference in temperatures explains the light-induced actuation shown in Fig. 1c. After thermomechanical programming, the red LED illumination induced downward bending (i.e., *n*-shape) owing to the selective light absorption only in blue SMP fibers. The temperature of the blue SMP fibers increased above T_g_, and the elongated blue fibers softened and shortened to the original length. The temperature of the yellow fibers remained relatively unchanged (i.e., below T_g_); thus, the yellow fibers retained their elongated length. This difference between the blue and yellow fibers induced the bending of the multilayer composite. The blue LED illumination induced the heating in the yellow fibers; thus, the yellow fibers also reverted to their original length. Then, the entire structure reverted to its initial flat shape as shown in Fig. 1c. This multicolor structure can be reused for another cycle of actuation after thermomechanical elongation.

**Figure 3:**
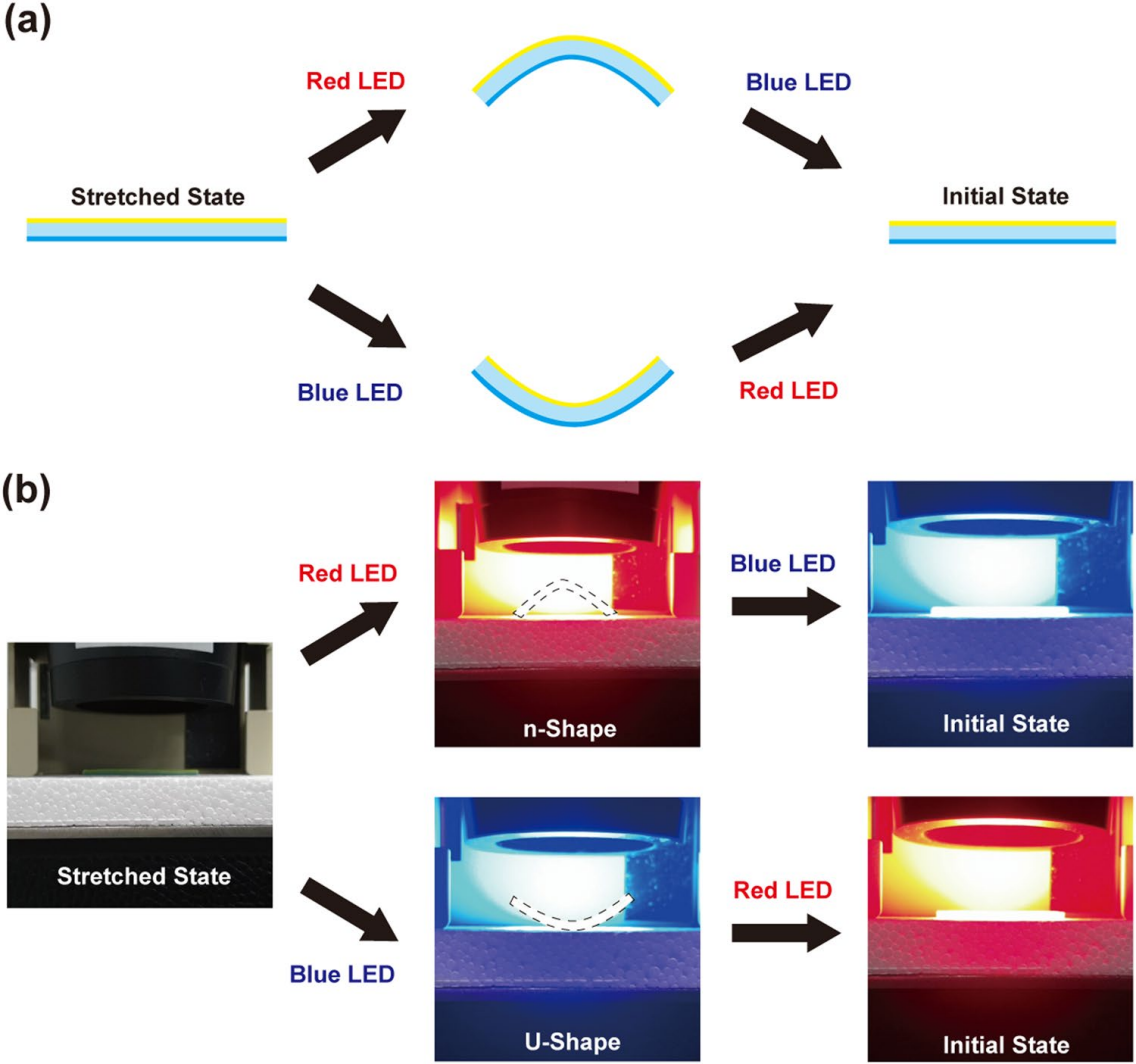
Bending behavior of the multicolor sample. A thermomechanically programmed structure bends to a n-shape under red illumination. After bending, the structure can recover to an initial flat state with blue illumination. In case of illuminating blue light first, the structure bends to a U-shape. It can also recover to the initial state with red-light illumination. (**a**) is the schematic for dual-step actuation, while (**b**) is the experimental result. See also Videos 1–4.

This color-dependent selective heating and actuation behavior also suggests that we can induce actuation into different shapes by controlling the illumination sequence of different colors of light. Figure 3 presents a simple example. Figure 3a,b show a schematic and an experimental result, respectively (see also Videos 1–4). When red light was first applied, the blue SMP fibers placed at the bottom of the printed structure recovered, whereas the yellow SMP fibers at the top remained rigid. Thus, the entire structure deformed into an *n*-shape.

Applying blue light later caused the entire structure to retain its initial flat state. In contrast, when blue light was first applied, the yellow SMP fibers at the bottom recovered first, whereas the blue SMPs remained rigid. Therefore, the entire structure was bent upward (i.e., deformed into a *U*-shape). Applying red light later caused the entire structure to retain its initial flat state. However, when the structure was heated in hot water (instead of selective heating with colored light), the change in shape of our sample was insignificant (data is not shown here). In the hot water, both blue and yellow SMPs recovered at the same rate, and the entire structure shrank to its original length but remained flat (i.e., no shape change occurred).

**Figure 4:**
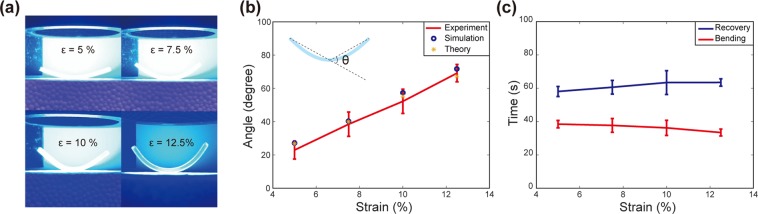
Bending and recovery under blue-light illumination. (**a**) Experiments with different programming strains. (**b**) Maximum bending angles from experiments, simulations, and analytical theory. The experiment was repeated 5 times for each strain, and the error bars were obtained from the standard deviation. (**c**) The measured time for maximum bending (red) and recovery to the initial state (blue).

To investigate the bending behavior, we performed thermomechanical programming with different strains and observed the resulting bending with blue LED illumination (Fig. 4). Light-activation experiments were conducted for samples with strains of 5%, 7.5%, 10%, and 12.5%; refer to Fig. 4a. Selective heating of yellow SMP fibers under blue LED illumination caused the entire structure to bend into a *U*-shape. Photographs of these experiments are shown in Fig. 4a. We notice that larger strains led to larger bending angles. In Fig. 4b, the maximum bending angle is plotted with respect to programming strain (red line). The maximum bending angle increased as the strain increased. Figure 4c shows the time taken to reach the maximum bending angle (red curve). The required time was almost the same for all the strains, but it decreased slightly as the strain increased. The slight decrease in the time can be attributed to the different heating rates of the SMP fibers with varying strains because the structures with larger strains became thinner. The temperature of thinner SMPs can increase faster.

Because of the heat transfer from the yellow to blue SMP fibers, the bent structure eventually became flat again under blue LED illumination; refer to Video 5. The blue line in Fig. 4b represents the measured time consumed for the full recovery of the flat structure. For all the programming strains, the full-recovery time was approximately 60 s. This implies that the temperatures of both the yellow and blue SMP fibers significantly exceeded T_g_ in 60 s, and both fibers retained their original lengths. This indicates that we must adjust the duration of illumination to control the bending behavior^29^.

**Figure 5:**
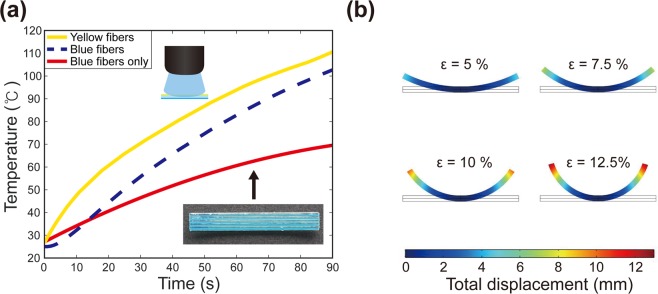
(**a**) The measured temperature change of the multicolor structure. The yellow solid line is the temperature of yellow SMP fibers, whereas the blue dashed line indicates the temperature of blue SMP fibers obtained from heat transfer simulations. The red solid line is the measured temperature of blue SMP fibers in a control sample that contains blue SMP fibers only. (**b**) Results of solid-mechanics simulations. The color bar indicates the total displacement measured from the bottom plane.

To understand the bending behavior further, we considered the temperature changes of yellow and blue SMP fibers under blue LED illumination (Fig. 5). In the case of yellow fibers at the upper layer, we directly measured the surface temperature of our multicolor sample using an IR imaging camera. Because the absorption of blue LED light by yellow fibers is strong, we could determine the temperature from the maximum-temperature spot; note the solid yellow line in Fig. 5a. The temperature of the blue fibers at the lower layer was estimated via heat-transfer simulations (COMSOL Multiphysics). In the simulations, the experimentally measured temperatures for yellow fibers were used, and the thermal-conductivity values for printing materials were obtained from the literature^32,33^. We found that air cooling does not change our simulation result much (see Supplementary Fig. S1). Heat can be gradually transferred from the yellow fibers to the blue fibers; thus, the temperature of the blue fibers increases over time; refer to Appendix I for simulation snapshots. Figure 5a shows the temperature change of the yellow (measured) and blue (estimated) SMP fibers in our sample. The dotted blue line represents the result of the heat-transfer simulation. Because the blue SMP fibers partially absorbed the blue light, the temperature of the blue fibers can increase by this direct absorption too. To investigate the effect of direct absorption, we prepared a control sample with only blue SMP fibers in a transparent matrix material (see the inset in Fig. 5a). Figure 5a also shows the measured temperature for this control sample under blue LED illumination (red solid line). The rise in temperature due to direct blue-light absorption was significantly smaller than that due to heat transfer. Thus, it shows that the dominant factor causing the temperature increase in the blue fibers at the lower layer was the heat transferred from the yellow fibers at the top layer.

We also simulated the bending behavior of our multilayer sample (COMSOL Multiphysics, Solid Mechanics Module). These simulations were conducted using the temperature data in Fig. 5a and the measured modulus and recovery ratio of the SMPs; refer to Appendix II and III. Figure 5b shows the simulation results for different programming strains. The bending angles from these simulations are presented in Fig. 4b (blue circles) and were found to agree well with the experimental results presented in Fig. 4b (red lines).

We also performed analytical model calculations to analyze our bending experiments. The radius *r* of the bending curvature was obtained using Timoshenko’s bilayer beam theory^34,35^:

1 $$r=\frac{{\rm{t}}\cdot \left[3\cdot {(1+m)}^{2}+(1+m\cdot n)\cdot \left({m}^{2}+\frac{1}{m\cdot n}\right)\right]}{6\cdot {(1+m)}^{2}\cdot \varepsilon },$$

where *t* represents the total thickness, *m* represents the thickness ratio between the two layers, *n* represents the modulus ratio between the two layers, and *ε* represents the strain difference between the two layers. See Appendix IV for the derivation of this equation. *t* and *m* are determined by the sample geometry, and *n* and *ε* can be estimated using the data in Fig. 5a and the measured SMP parameters. For instance, in the case of 7.5% strain, the maximum bending takes 38 s (Fig. 4b). As shown in Fig. 5a, the temperatures of the yellow and blue fibers become 77.2 and 63.7 °C at this time, respectively. Then, at these temperatures, the moduli of the yellow and blue fibers are approximately 12.7 and 76.6 MPa, respectively (Appendix II). Note that the modulus of the transparent matrix material is significantly lower (i.e., ~0.5 MPa). The effective modulus *M*_*eff*_ of a composite material in the isostrain condition can be determined as follows:

2 $${M}_{eff}={V}_{m}\cdot {M}_{m}+{V}_{f}\cdot {M}_{f},$$

where *V*_*m*_ and *V*_*f*_ represent the volume fractions of matrix and fiber materials, respectively, and *M*_*m*_ and *M*_*f*_ represent the moduli of matrix and fiber materials, respectively. Then, the effective modulus of the upper half layer (containing yellow fibers) becomes ~2.3 MPa and that of the lower half layer (containing blue fibers) becomes ~11.6 MPa. Thus, we obtain the modulus ratio between the two layers as *n* ~ 5.1.

The measured recovery ratio of the blue SMPs was 25.2% at 63.7 °C (Appendix III). Assuming that the upper-half layer containing yellow fibers is shortened to its original length at the maximum bending, we obtained the strain difference between the two layers as *ε* ~ 0.0374. From these *n* and *ε* values, we calculated the bending radius as *r* ~ 48.6 mm. For other strains, we can perform the same calculations. The corresponding bending angles for different programing strains are plotted in Fig. 4b (orange asterisks). The analytical results agree well with the experimental results. Clearly, the maximum bending angle increases as the programming strain increases. Because the difference in length between the blue and yellow SMP fibers causes the structure to bend, a larger length difference yields a larger maximum bending angle. We also note that the viscoelasticity of SMPs can complicate the analysis^36–38^. We measured the recovery ratio of SMPs over time (Appendix III). Because this recovery ratio is affected by the time-dependent relaxation of polymers, it includes the viscoelastic response of SMPs. The measured recovery ratio was used in our analytic modeling, as described above. In our work, viscoelasticity was considered in this way, and we found that our analysis agrees well with experiments.

In Fig. 4c, we discussed that bent structures eventually became flat again because of heat transfer under continuous light illumination. Similar heat transfer would occur even after the light source is turned off. For example, in Fig. 3b, we observed that the bent structure relaxed a little (i.e., the bending angle was reduced back a little) after light was turned off. The residual heat in the structure could cause this relaxation to happen. To resolve this issue, a thermal insulating layer could be potentially included inside the multicolor composite to block the heat flow.

**Figure 6:**
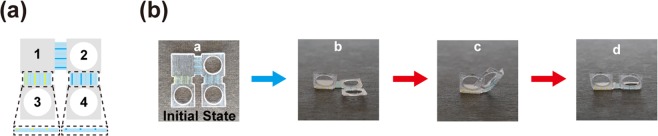
Multicolor hinged structure for multistep actuation. **(a**) Schematic for the multicolor hinged structure. (**b**) Example of multistep actuation. This hinged structure can transform into different 3D shapes depending on the color of light and duration of illumination. See also Videos 6–8.

Finally, we demonstrate a multicolor hinged structure for multistep actuation. Here, we used colored SMP fibers in the hinges and controlled the sequential shape transformation according to the color of light; refer to Fig. 6. As shown in Fig. 6a, there were four panels in the structure. Instead of having yellow and blue SMP fibers in the same hinge, we used transparent SMP (Veroclear) fibers with either yellow or blue fibers in a transparent matrix (colored blue in the schematic). The transparent SMPs are colored gray in the schematic. We also used the transparent SMP in the rigid panel of the structure. The two hinges between panels 1 and 2 and between panels 2 and 4 were composed of blue and transparent SMP fibers. The hinge connecting panels 1 and 3 consisted of yellow and transparent fibers. Hinges containing yellow SMPs primarily respond to blue light and those containing blue SMPs respond to red light more strongly; refer to Fig. 2a. In this design, the length of each rigid panel was 5 mm. The length of the SMP fibers was 2.5 mm, and their width and thickness were 0.2 mm. During thermomechanical programming, all the hinges were elongated in hot water and cooled equally. We formed holes on panels 2–4 to reduce the weight. To realize rapid transformation, we focused the LED light onto our structure using a focal lens. Our structure transformed within 10 s under LED illumination.

This hinged structure can be transformed into multiple different shapes depending on the color sequence and duration of illumination. Figure 6b shows an example (see also Videos 6–8). With blue light illumination, the yellow SMP fibers in the hinge between panels 1 and 3 relaxed, and panel 3 rose (State b). With red illumination, the other two hinges bent as well; thus, we obtained another shape (State c). With additional red illumination, the hinge between panels 1 and 2 straightened because of the heat transfer to the transparent SMP fibers (colored gray). However, the other hinge between panels 2 and 4 remained bent because this hinge was slanted and received less light by the illumination from the top. Thus, by controlling the color of light and duration of illumination, we could transform the hinged structure into different shapes. More designs could be further developed to realize various structural changes.

## Conclusions

We demonstrated multicolor 4D printing of SMP composites and remote actuation via color-dependent selective heating. We experimentally investigated the light absorption and temperature changes in colored SMPs and observed clear differences in the color-dependent temperature changes. The bending-angle changes were examined both experimentally and theoretically. For theoretical modeling, we performed both numerical simulations and analytic calculations to explain the bending-angle dependence on the programming strain. Finally, we demonstrated a multicolor hinged structure, where multistep actuation was controlled by the color of light and duration of illumination. SMPs can be reused by conducting thermomechanical programming again and their response temperatures can be adjusted via material synthesis or by dynamic mixing during 3D printing. Moreover, 4D printing can enable the fabrication of complex, multicolor geometries for tailored responses. Therefore, multicolor 4D printing of SMP composites have unique merits for light-induced structural changes and remote actuation.

**Appendix I**: Heat transfer simulation

**Figure 7:**
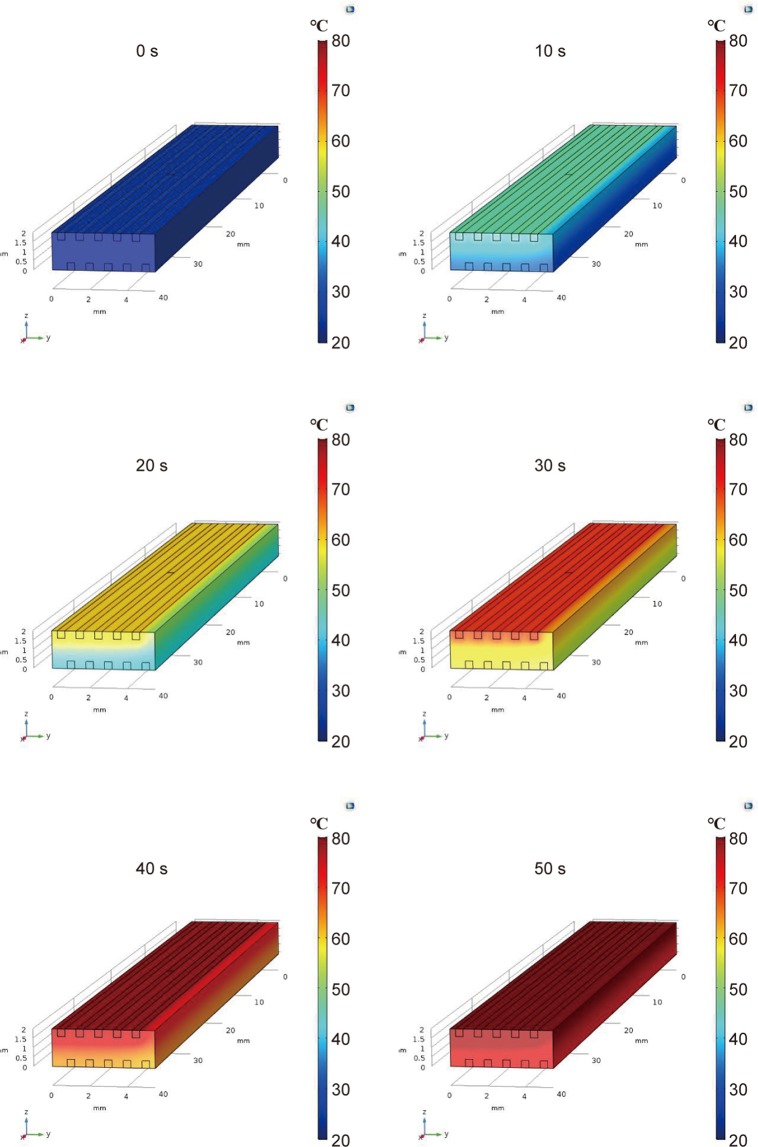
Snapshots from heat transfer simulation (COMSOL Multiphysics).

Figure 7 shows the results from heat transfer simulations; the snapshots show heat transfer and temperature changes over time. The experimentally measured temperatures were used for yellow fibers. The temperature of the blue fibers increases over time because of heat transfer from the yellow fibers to the blue fibers.

**Appendix II**: Modulus measurements with dynamic mechanical analysis (DMA)

**Figure 8:**
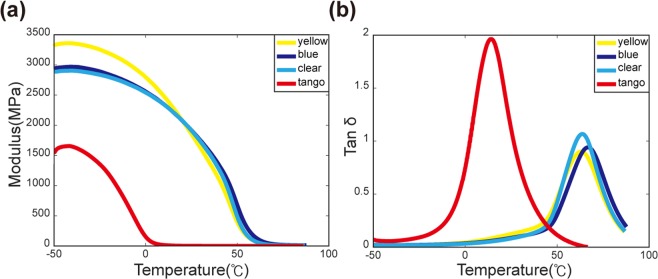
(**a)** Storage modulus and (**b**) Loss tangent obtained with dynamic mechanical analysis (DMA) measurements.

Figure 8 shows the storage modulus and loss tangent of the SMPs and the matrix material that we used in our work. The DMA sample (size: 10 mm × 3  mm × 1  mm) was 3D-printed using Stratasys J750 and mounted on a DMA machine (TA Instruments, Q800). A preload of 0.01 N was imposed during measurements to prevent the sample from bending. The equilibrium was initially set at −50 °C for 5 minutes, and then temperature was increased to 90 °C with a rate of 3 °C/min. During measurements, the strain oscillated with a frequency of 1 Hz at 0.1% peak amplitude. Three colorful SMP materials (yellow, blue, clear) have nearly equal modulus values above the room temperature. This helps our multi-color composite structures remaining flat after thermomechanical programming. The peak of tanδ shows glass transition temperatures (T_g_). The T_g_’s of yellow, blue, transparent SMPs are 63.2 °C, 66.7 °C, and 63.5 °C, respectively.

**Appendix III**: Recovery ratio measurements

The recovery ratio of the SMP we used was again measured using DMA. The measuring sample was 20 mm × 6  mm × 1  mm in size. The recovery ratio was measured after the following thermomechanical programming: Temperature was first increased to 90 °C by a rate of 2 °C/min and then maintained at 90 °C isothermally for 5 minutes until the sample reached the same temperature. Then, the sample was stretched with a 10% strain with a rate of 0.5%/min at 90 °C. After stretching, the temperature was decreased to 20 °C by a rate of 2 °C/min. Then, the force was gradually made zero with the same rate as the stretching case.

**Figure 9:**
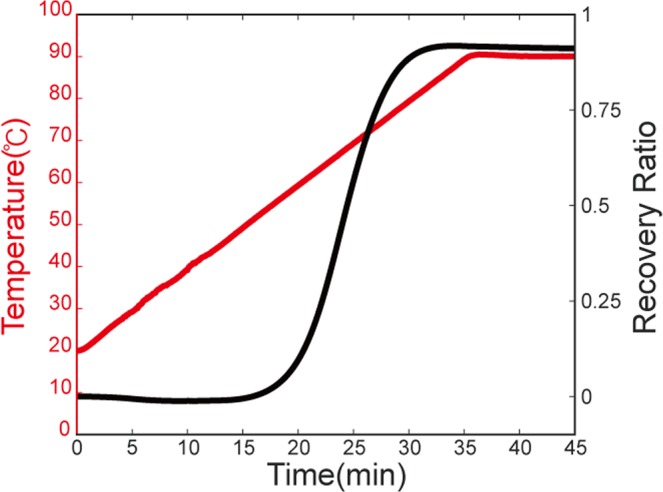
Recovery ratio measurement of the yellow SMP.

To measure the shape-memory recovery ratio, the temperature was again increased to 90 °C by the rate of 2 °C/min and maintained isothermally for 10 minutes (red curve in Fig. 9). Then, the recovery ratio was determined from 1 – *ε*(t)/*ε*(0), where *ε*(t) is the current strain value of the SMP and *ε*(0) is the strain value just before the recovery process. The maximum recovery ratio of the SMP was about 92%. The shape fixity ratio of SMPs can be also estimated from parameters obtained in DMA measurements (see Supplementary Figure Fig. S2). The measurement result is summarized in Table 1.

**Table 1:** Shape fixity ratio of SMPs.

Maximum strain before unloading	Strain after unloading	Fixity ratio
9.62%	9.5%	98.75%

**Appendix IV**: Bilayer beam theory

**Figure 10:**
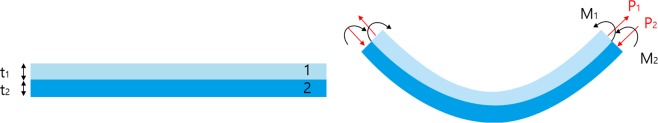
Schematic for a bilayer beam.

The radius of the bending curvature can be obtained from Timoshenko’s beam theory^34,35^, as follows. In order to satisfy the equilibrium, the following two equations should be satisfied (refer to Fig. 10 for the bilayer geometry and relevant parameters):

$${P}_{1}={P}_{2}=P$$

$$\frac{Pt}{2}={M}_{1}+{M}_{2}$$

The bending moment *M* of each beam can be expressed using the modulus (*E*), the second moment of area (I), and the radius *r* of bending curvature.

$$\begin{array}{c}{M}_{1}=\frac{{E}_{1}\cdot {I}_{1}}{r}\\ {M}_{2}=\frac{{E}_{2}\cdot {I}_{2}}{r}\end{array}$$

Then,

$$\frac{Pt}{2}=\frac{{E}_{1}{I}_{1}+{E}_{2}{I}_{2}}{r}$$

The strain difference between two layers can be expressed by above terms:

$${\rm{\varepsilon }}=\frac{{P}_{1}}{{E}_{1}{t}_{1}}+\frac{{P}_{2}}{{E}_{2}{t}_{2}}+\frac{{t}_{1}}{2r}+\frac{{t}_{2}}{2r}$$

Eliminating P terms using the equation just above with the equation of second moment of area, we obtain

$${I}_{1}=\frac{{t}_{1}^{3}}{12},\,{I}_{2}=\frac{{t}_{2}^{3}}{12}$$

Finally, we obtain the following expression for the radius of bending curvature.

$${\rm{r}}=\frac{{\rm{t}}\cdot \left[3\cdot {(1+m)}^{2}+(1+m\cdot n)\cdot \left({m}^{2}+\frac{1}{m\cdot n}\right)\right]}{6\cdot {(1+m)}^{2}\cdot \varepsilon }$$

where *t* is the total thickness, *m* is the thickness ratio between two layers, *n* is the modulus ratio between two layers, and *ε* is the strain difference between two layers. This is the Eq. (1) in the main text. From the radius, we can estimate a corresponding bending angle.

## Supplementary information

Supplementary Information.

Supplementary Video 1

Supplementary Video 2

Supplementary Video 3

Supplementary Video 4

Supplementary Video 5

Supplementary Video 6

Supplementary Video 7

Supplementary Video 8

## Data Availability

The raw data and the processed data required to reproduce the findings in the current study are available from the corresponding author on reasonable request.
