# Supplementary material for: Multicolor 4D printing of shape-memory polymers for light-induced selective heating and remote actuation
Source: Sci Rep. 2020 Apr 10;10:6258. doi: 10.1038/s41598-020-63020-9 (PMC7148339; doi:10.1038/s41598-020-63020-9)
Supplement: Supplementary file 1 — Supplementary Information. [file 41598_2020_63020_MOESM1_ESM.pdf]

Supplementary Information for

Multicolor 4D printing of shape-memory polymers for light-induced selective heating and remote actuation

Hoon Yeub Jeong[1], Byung Hoon Woo[1], Namhun Kim[2,*] & Young Chul Jun[1,†]

[1]School of Materials Science and Engineering, Ulsan National Institute of Science and Technology (UNIST), Ulsan 44919, Republic of Korea

[2]School of Mechanical, Aerospace and Nuclear Engineering, UNIST, Ulsan 44919, Republic of Korea

- Fig. S1: Heat transfer simulations with and without air convection
- Fig. S2: Sequence of DMA measurements

Supplementary Figure S1

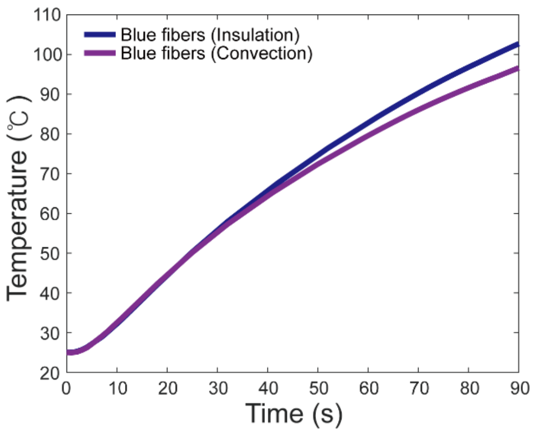

Fig. S1. Heat transfer simulations with and without air convection. The purple line is the case including air cooling (i.e. air convection). We found that air cooling does not change our simulation result much. Note that the actual shape change in our bending experiments took 30~40 seconds. Up to this time, the air cooling does not make much difference in simulated temperatures – e.g., the temperature difference is about 1 ℃ around 40 s. Based on air convection simulations, we think that air cooling does not affect much the maximum bending angle of our sample.

The heat transfer simulations were conducted with the COMSOL Multiphysics (heat transfer module). Using the laminar flow mode, we simulated the temperature of the blue shape memory fibers as the yellow fibers heated up over time. To investigate the effect of air cooling, air domains with rectangular shapes were added around the sample; the five sides of the sample were set to open boundary conditions except the surface beneath the sample. A thermal insulation condition was applied to this bottom surface, because the printing sample was placed on a Styrofoam in experiments for thermal insulation.

Supplementary Figure S2

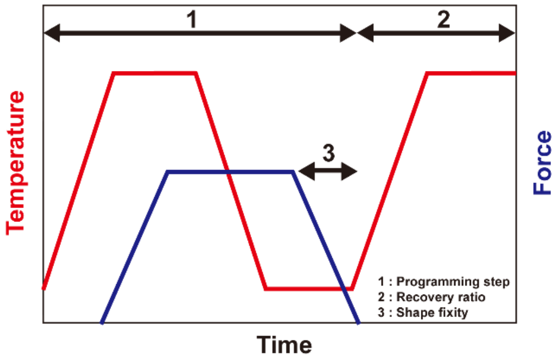

Fig. S2. Sequence of DMA measurements. Following the programming step in the period 1, the recovery ratio was measured in the period 2 (see Fig. 9 in the main text). The shape fixity can be estimated from measured parameters in the period 3. The estimated shape fixity is summarized in Table 1 in the main text.
